# 5-*O*-methylcneorumchromone K Exerts Antinociceptive Effects in Mice via Interaction with GABAA Receptors

**DOI:** 10.3390/ijms22073413

**Published:** 2021-03-26

**Authors:** Luiza Carolina França Opretzka, Humberto Fonseca de Freitas, Renan Fernandes Espírito-Santo, Lucas Silva Abreu, Iura Muniz Alves, Josean Fechine Tavares, Eudes da Silva Velozo, Marcelo Santos Castilho, Cristiane Flora Villarreal

**Affiliations:** 1 Laboratório de Farmacologia e Terapêutica Experimental, Faculdade de Farmácia, Universidade Federal da Bahia, Salvador CEP 40 170-115, Brazil; luizacfo@ufba.br (L.C.F.O.); hffreitas@ufba.br (H.F.d.F.); r.fernandes88@hotmail.com (R.F.E.-S.); iuratr4@gmail.com (I.M.A.); euvelozo@ufba.br (E.d.S.V.); castilho@ufba.br (M.S.C.); 2 Laboratório de Engenharia Tecidual e Imunofarmacologia, Instituto Gonçalo Moniz, Fundação Oswaldo Cruz, Salvador CEP 40 296-710, Brazil; 3 Instituto de Pesquisa em Fármacos e Medicamentos, Universidade Federal da Paraíba, João Pessoa CEP 58 050-585, Brazil; lucas.abreu@ltf.ufpb.br (L.S.A.); josean@ltf.ufpb.br (J.F.T.)

**Keywords:** chromone, analgesic, GABA, docking

## Abstract

The proper pharmacological control of pain is a continuous challenge for patients and health care providers. Even the most widely used medications for pain treatment are still ineffective or unsafe for some patients, especially for those who suffer from chronic pain. Substances containing the chromone scaffold have shown a variety of biological activities, including analgesic effects. This work presents for the first time the centrally mediated antinociceptive activity of 5-O-methylcneorumchromone K (5-CK). Cold plate and tail flick tests in mice showed that the 5-CK-induced antinociception was dose-dependent, longer-lasting, and more efficacious than that induced by morphine. The 5-CK-induced antinociception was not reversed by the opioid antagonist naloxone. Topological descriptors (fingerprints) were employed to narrow the antagonist selection to further investigate 5-CK’s mechanism of action. Next, based on the results of fingerprints analysis, functional antagonist assays were conducted on nociceptive tests. The effect of 5-CK was completely reversed in both cold plate and tail-flick tests by GABA_A_ receptor antagonist bicuculline, but not by atropine or glibenclamide. Molecular docking studies suggest that 5-CK binds to the orthosteric binding site, with a similar binding profile to that observed for bicuculline and GABA. These results evidence that 5-CK has a centrally mediated antinociceptive effect, probably involving the activation of GABAergic pathways.

## 1. Introduction

Pain is a symptom associated with a variety of pathological states, frequently it is the first or the most important manifestation of a disease. However, sometimes pain can turn into a disease itself. When pain exceeds its biological function of protection and becomes chronic it negatively impacts both day-to-day life and mental health [1,2]. The proper pharmacological control of pain is an unsolved challenge for patients and health care providers. The ongoing treatment is primarily based on the use of opioid analgesics and non-steroidal anti-inflammatory drugs (NSAID). Even though those pharmacological classes are among the most widely used medications, they still are ineffective or unsafe for some patients, especially for those who suffer from chronic pain [3,4]. Yet, there are few drug development efforts that focus on targeting novel pathophysiological mechanisms to supply the treatment of patients who do not respond to available drugs [5,6].

Chromones are a class of naturally occurring substances, widely distributed throughout nature, which has been recognized as a privileged structure for drug discovery since it has been associated with a variety of biological activities such as antifungal, antidiabetic, antiviral, and anti-hypertensive [7,8]. In addition, several molecules containing the chromone moiety display anti-inflammatory [9] and analgesic [10,11,12] activities. Chromone-like compounds were found to be opioid ligands [13], but their analgesic activities have also been associated with other types of receptors [10,14]. Some could also potentially interact within the endogenous analgesia system by acting as serotonin receptor ligands [15,16]. Besides, these compounds are considered promising for pharmaceutical development because they possess low toxicity and are available in the human diet, due to their wide distribution among edible plants [17].

Previously, we reported that a chromone compound isolated from *Dictyoloma vandellianum* (Adr. Juss), named 5-*O*-methylcneorumchromone K (5-CK), has consistent anti-inflammatory activity and favorable pharmacokinetic properties, an interesting feature for drug development [18]. Based on the analgesic properties already described for chromones, the present work was designed to investigate whether 5-CK has intrinsic analgesic properties and to investigate its mechanism of action using in vivo and in silico approaches.

## 2. Results

### 2.1. Assessment of Antinociceptive Activity: Screening with Formalin Test

**Figure 1:**
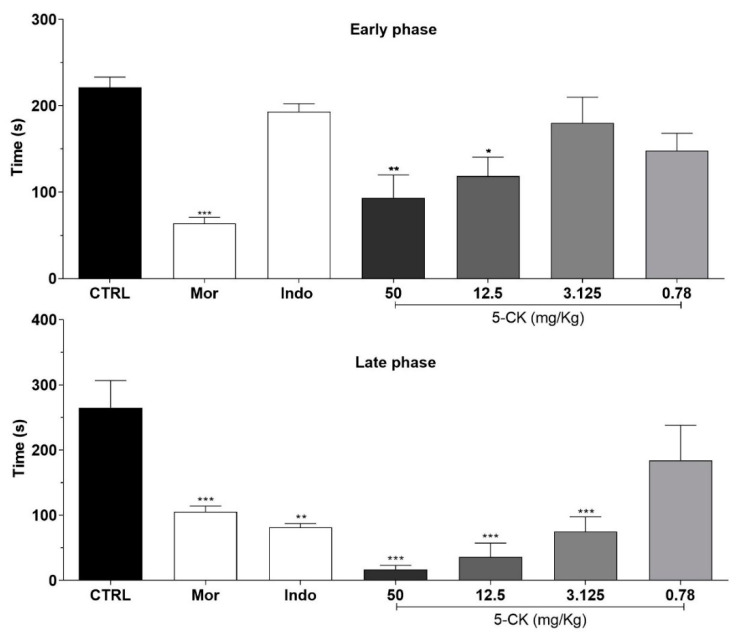
Effects of the systemic treatment with 5-CK in the early and late phases of the formalin test. Mice were treated with 5-CK (50–0.78 mg/Kg) or vehicle (CTRL, 50% propylene glycol in saline; control group) by intraperitoneal (ip.) route 40 min before formalin (injected at time zero). Morphine (Mor; 5 mg/Kg, ip.) and indomethacin (Indo; 10 mg/Kg, ip.) were used as reference drugs. Data are expressed as means ± SEM; *n* = 6 mice per group. * Significantly different from the control group (*p* < 0.05). ** Significantly different from the control group (*p* < 0.01). *** Significantly different from the control group (*p* < 0.001) as determined by ANOVA followed by Tukey’s test.

To assess the antinociceptive activity of 5-CK the formalin test was employed, a screening tool widely used to identify analgesic molecules. Figure 1 shows the effect of different doses of 5-CK on the formalin test in mice. Overall, mice pretreated with the vehicle spent more time performing nociceptive behaviors than mice pretreated with 5-CK or the gold standard drugs, morphine and indomethacin. In the early phase of the test, the pretreatment with 5-CK at 50 and 12.5 mg/Kg diminished the nociception caused by formalin (*p* < 0.05), indicating a potential analgesic activity. The late phase of the test was inhibited by 5-CK at doses up to 3.125 mg/Kg (*p* < 0.001). As expected, morphine inhibited both the early and late phases (*p* < 0.001), while indomethacin inhibited just the late phase of the formalin test (*p* < 0.001). These results suggest a potential analgesic activity of 5-CK as it exhibits a profile similar to that of morphine. There were no signs of motor impairment in mice from the different experimental groups, as assessed by the rota-rod test (data not shown), corroborating the antinociception suggested by the nociceptive test.

### 2.2. Further Characterization of 5-CK’s Antinociceptive Activity

**Figure 2:**
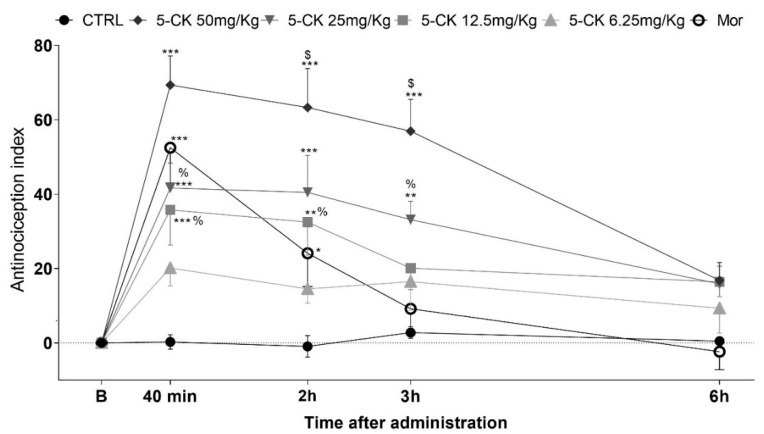
Antinociceptive effect of 5-CK on the tail flick test. Mice were treated by intraperitoneal (ip.) route 40 min before the test. For the dose−response analysis, the effects of increasing doses (6.25 to 50 mg/Kg) were tested. To evaluate the time-course of the antinociceptive effect, the thermal nociceptive threshold was measured before and up to 24 h following administration of 5-CK, vehicle (CTRL, 50% propylene glycol in saline; control group) or morphine (Mor; 5 mg/Kg, ip.), the reference drug. Data are expressed as mean ± SEM; *n* = 6 mice per group. ^$^ Significantly different from morphine group (*p* < 0.001) ^%^ Significantly different from 5-CK 50 mg/Kg group (*p* < 0.05). * Significantly different from the control group (*p* < 0.05). ** Significantly different from the control group (*p* < 0.01). *** Significantly different from the control group (*p* < 0.001). Two-way ANOVA followed by Bonferroni’s test.

**Figure 3:**
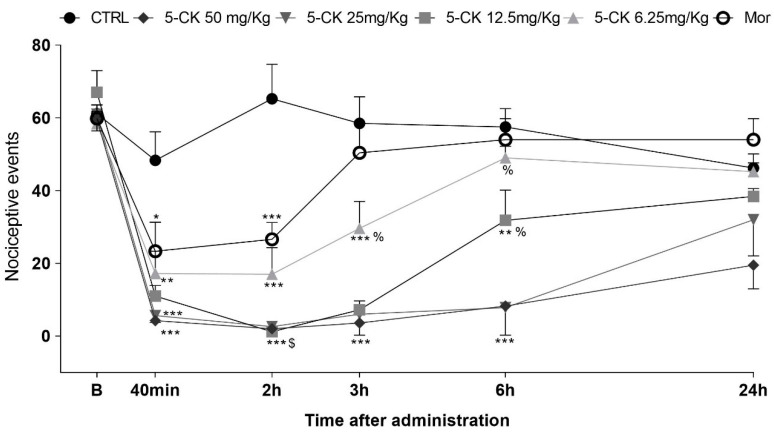
Antinociceptive effect of 5-CK on the cold plate test. Mice were treated by intraperitoneal route 40 min before the test. For the dose−response analysis, the effects of increasing doses (6.25 to 50 mg/Kg) were tested. To evaluate the time-course of the antinociceptive effect, the thermal nociceptive threshold was measured before (B) and up to 24 h following administration of 5-CK, vehicle (CRTL, 50% propylene glycol in saline; control group) or morphine (Mor; 5 mg/Kg), the reference drug. Data are expressed as mean times ± SEM; *n* = 6 mice per group. ^$^ Significantly different from morphine group (*p* < 0.001). ^%^ Significantly different from 5-CK 50mg/Kg group (*p* < 0.05). * Significantly different from the control group (*p* < 0.05). ** Significantly different from the control group (*p* < 0.01). *** Significantly different from the control group (*p* < 0.001). Two-way ANOVA followed by Bonferroni’s test.

To confirm and characterize the antinociceptive activity of 5-CK, tail-flick (Figure 2) and cold plate tests (Figure 3), well-established assays to central analgesics, were conducted. The dose-response relationship and time course of antinociception were established. Intraperitoneal (ip.) administration of 5-CK enhanced the latency of the tail withdrawal reflex at doses of 50, 25 and 12.5 mg/Kg (*p* < 0.001), which can be perceived as an increase of the antinociception index in the tail flick test (Figure 2). This effect occurred in a dose-dependent manner (*p* < 0.05). The antinociception started 40 min after the administration of 5-CK and lasted up to 3 h after its administration for the doses of 50 mg/Kg and 25 mg/Kg (*p* < 0.001; *p* < 0.01). Importantly, the antinociceptive effect of 5-CK in the tail flick test was more efficacious (2 h; *p* < 0.001) and lasted longer when compared to morphine (5 mg/Kg, ip.), the gold standard drug in this test.

The administration of 5-CK also reduced the nociceptive behavior of mice in the cold plate test in a consistent, dose-dependent, and long-lasting manner (Figure 3). The pretreatment with 5-CK decreased the number of nociceptive events at all tested doses (6.25−50 mg/Kg; *p* < 0.01). This antinociceptive effect was dose-dependent starting 40 min after the administration of 5-CK. The doses of 50, 25 and 12.5 mg/Kg kept their effects up to 6 h after the administration. As expected, the gold standard morphine (5 mg/Kg, ip.) decreased the number of nociceptive events for up to 2 h (*p* < 0.001) after administration. In accordance with the tail flick test, the antinociceptive effect of 5-CK was longer-lasting and more efficacious than the one induced by morphine (2 h; *p* < 0.001). Taken together, these results corroborate the data from the formalin test and indicate that the antinociceptive effect of 5-CK is, at least in part, centrally mediated.

### 2.3. Investigation of the Contribution of the Opioid System in 5-CK-Induced Antinociception

**Figure 4:**
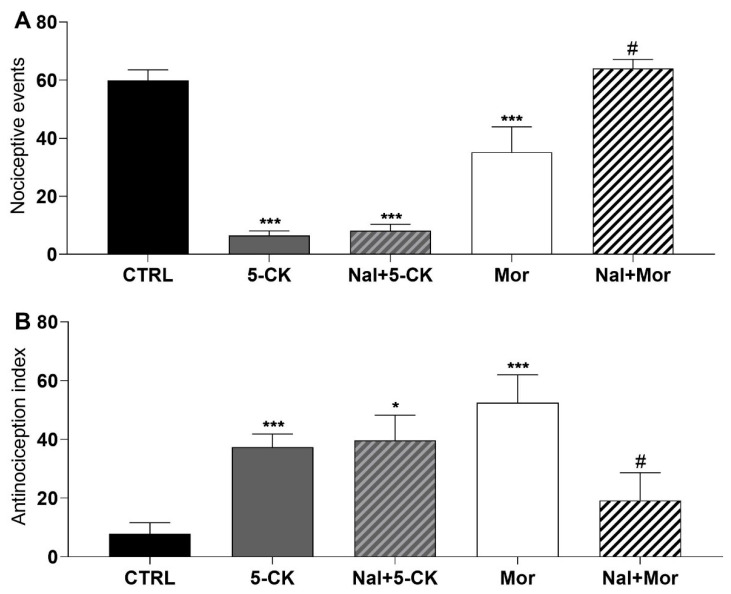
Effect of the pharmacological blockade of opioid receptors on the 5-CK-induced antinociception. Antagonism assays were performed on cold plate; and (**A**) tail flick; (**B**) tests with naloxone (Nal, 5 mg/Kg), a non-selective antagonist of opioid receptors. Naloxone was administered by intraperitoneal route (ip) 15 min before the administration of 5-CK (50 mg/Kg, ip) or morphine (Mor; 5 mg/Kg, ip). Data are expressed as means ± SEM; *n* = 6 mice per group. # Significantly different from Mor group (*p* < 0.05) * Significantly different from the control group (*p* < 0.05). *** Significantly different from the control group (*p* < 0.001). One-way ANOVA followed by Tukey’s test.

In order to investigate the possible mechanisms underlying 5-CK’s centrally mediated antinociception, functional antagonist assays were conducted. Initially, naloxone (a non-selective opioid receptor antagonist) was tested, since 5-CK showed an antinociceptive profile similar to that of morphine. Naloxone completely reversed the antinociceptive effect of morphine on cold plate (Figure 4A) and tail flick (Figure 4B) tests, as expected. On the other hand, the pre-treatment (15 min) with naloxone did not revert the antinociceptive effect induced by 5-CK on cold plate (Figure 4A) and tail flick (Figure 4B) tests, indicating that this system does not contribute to the mechanism of action of 5-CK.

### 2.4. Target Selection by Ligand Similarity Approach

**Figure 5:**
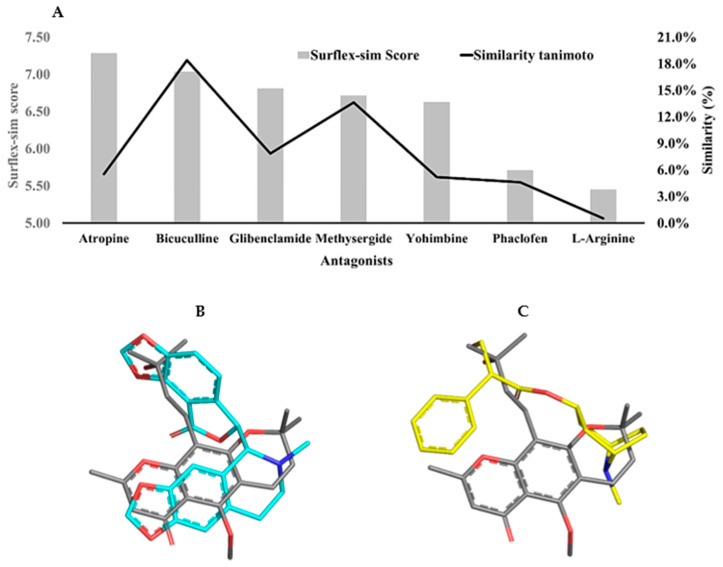
2D and 3D similarity analysis of 5-CK and antinociception-related antagonists. (**A**) Tanimoto coefficient (%) and morphological similarity score of 5-CK against antagonists of receptors and channels involved with the endogenous analgesia system. (**B**,**C**) 3D alignment of 5-CK (gray sticks) on atropine (yellow sticks) and bicuculline (cyan sticks), the two most similar ligands according to Surflex-Sim (morphological similarity). Atom coloring scheme — nitrogen: blue, oxygen: red, carbon: gray, yellow or cyan, as described above.

Topological descriptors (fingerprints) were employed to evaluate the chemical similarity between 5-CK and several antagonists of receptors and ion channels related to endogenous analgesia pathways (Supplementary Materials Figure S1), namely methysergide maleate (serotonergic receptor antagonist); yohimbine (alpha-2 adrenergic receptor antagonist); atropine (cholinergic receptor antagonist); bicuculline (gamma aminobutyric acid-A—GABA_A_ receptor antagonist); phaclofen (gamma aminobutyric acid-B—GABA_B_ receptor antagonist); L-arginine (precursor of nitric oxide); glibenclamide (ATP-sensitive potassium channel blocker). In general, the Tanimoto coefficients are quite low (<20%) and suggest that 5-CK’s target cannot be identified by this method. On the other hand, morphological analysis, which is a 3D method, suggests that 5-CK has a significant morphological similarity to atropine (7.28), bicuculline (7.03), and glibenclamide (6.81) (Figure 5A). Although 5-CK has a higher Surflex-sim score for atropine than bicuculline, a visual analysis suggests that 5-CK is better aligned with bicuculline (Figure 5B,C).

### 2.5. Investigation of the Contribution of Different Analgesic Pathways to 5-CK-Induced Antinociception Using Functional Antagonist Assays

**Figure 6:**
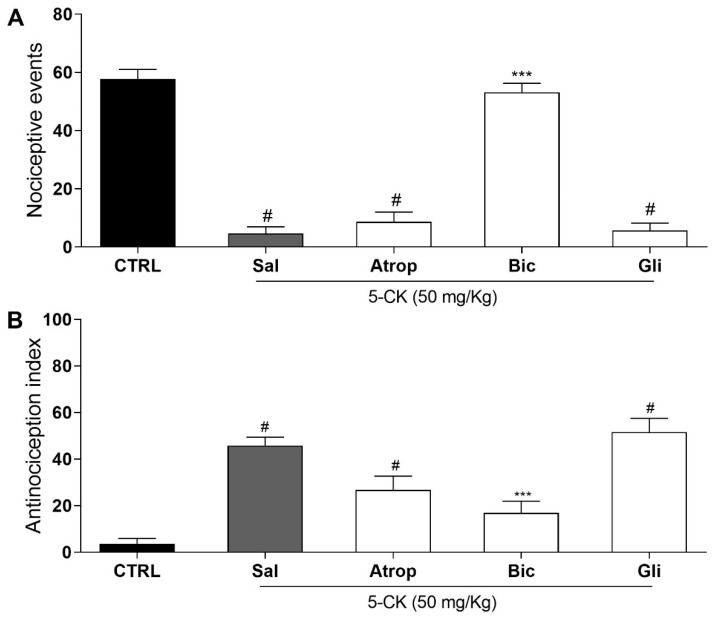
Assessment of possible mechanisms involved in the antinociceptive effect of 5-CK. Functional antagonism assays were performed on cold plate; and (**A**) tail flick; (**B**) tests using atropine (Atrop; 10 mg/Kg, 15 min before, cholinergic receptor antagonist), bicuculline (Bic; 1 mg/Kg, 15 min before, GABA_A_ receptor antagonist) or glibenclamide (Gli; ATP-sensitive potassium channel blocker, 2 mg/Kg, 30 min before). Mice were treated by intraperitoneal routes with antagonists or saline (Sal) before the administration of 5-CK (50 mg/Kg) and 40 min afterward were submitted to nociceptive tests. The control group (CTRL) was treated with vehicle (50% propylene glycol in saline) by intraperitoneal route 40 min before the test. Data are expressed as means ± SEM; *n* = 6 mice per group. # Significantly different from CTRL group (*p* < 0.05). *** Significantly different from Sal group (*p* < 0.001). One-way ANOVA followed by Tukey’s test.

Guided by in silico results, the antagonist selection was narrowed to further investigate 5-CK’s mechanism of action in vivo by performing functional antagonism assays on cold plate and tail-flick tests (Figure 6). The selected antagonists were the top-scored atropine, bicuculline, and glibenclamide. Intraperitoneal pretreatment with saline, atropine (cholinergic receptor antagonist, 10 mg/Kg ip., 15 min pretreatment), or glibenclamide (ATP-sensitive potassium channel blocker, 2 mg/Kg ip., 30 min pretreatment) did not modify 5-CK’s antinociceptive effect. However, pretreatment with GABA_A_ antagonist bicuculline (1 mg/Kg, ip., 15 min pretreatment; *p* < 0.001) completely reversed the 5-CK-induced antinociceptive effect in both cold plate and tail-flick tests. These results show the contribution of GABAergic pathways activation to 5-CK’s antinociception and corroborate the results provided by the flexible 3D alignment obtained with Surflex-sim.

### 2.6. Molecular Docking Studies

**Figure 7:**
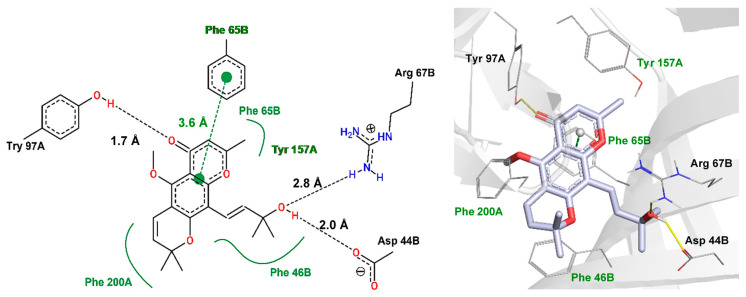
Interaction profile of 5-CK docked on bicuculline binding site (orthosteric binding site). The hydrogen bonds are displayed as black-dashed lines (two-dimensional (2D) view—**left**) or yellow lines (three-dimensional (3D) view—**right**), hydrophobic interactions as green lines (2D), and pi-stacking interaction as green-dashed line (2D). These interactions are not displayed in the 3D view, but the equivalent amino acids are labeled in green. The red and blue sticks (3D) represent oxygen and nitrogen atoms, respectively. Distances are measured in angstroms (Å). The interaction pattern was generated with Poseview (2D) and PLIP (3D) servers.

Cold plate and tail flick results suggest that 5-CK interacts with GABA_A_ receptors and morphological similarity analysis is compatible with 5-CK binding to the bicuculline binding sit. To shed light on the structural features that are required for this binding mode profile, a molecular docking protocol was carried out. Although, several GABA_A_ receptors are available on protein data bank (PDB) server, only a low-resolution structure (PDB: 3UHK, 3.75 Å) shows the binding coordinates for bicuculline. Due to the low resolution, this structure is not suitable for docking studies. Instead, a higher resolution GABA_A_ receptor structure (PDB: 6 × 3T, 2.55 Å) was morphed to the coordinates of the bicuculline-complexed structure [19] (Supplementary Materials Figure S2). The bicuculline binding site in the morphed structure (frame 25), (RMSD to 6HUK = 0.43 Å) is similar to the one observed in the low-resolution structure and the redocking of bicuculline affords a pose (best ranked) with the same binding profile described in cryo-EM structure (RMSD = 0.37 Å) (Supplementary Materials Figure S3). Therefore, it seems that GOLD default docking parameters and the morphed structure are suitable to predict how 5-CK will bind within the bicuculline site (Figure 7 and Supplementary Materials Figure S4).

According to docking results, 2-methylchromone moiety performs a π-π-stacking interaction to Phe65B (3.6 Å). Additional hydrophobic interactions of this moiety, along with dihydro-2*H*-pyran ring are found with Try157A, Phe46B, Phe65B, and Phe200B. The carbonyl group hydrogen-bonds to Try97A, whereas the 2-penten-4-ol moiety interacts with Arg67B and Asp44B which, to our knowledge, have not been explored in experimental studies.

## 3. Discussion

Molecules containing the chromone moiety exhibit a wide variety of biological activities, including anti-inflammatory and analgesic [9]. Although their analgesic effect is mostly associated with an anti-inflammatory activity, it has also been linked to simultaneous peripheral and central antinociceptive mechanisms [20,21,22,23]. In a previous work from our group, the natural chromone 5-CK showed anti-inflammatory properties [18]. Here we demonstrate that 5-CK has intrinsic analgesic properties, not directly linked to the control of inflammation, and which involves the participation of the central nervous system probably mediated by GABA_A_ receptors.

To assess the antinociceptive activity of 5-CK the formalin test was initially employed as a widely recognized screening test to analgesics. During the test, the nociceptive response of mice to formalin injection is timed and shows a biphasic pattern with an early and a late phase. The early phase is mostly related to direct activation of nociceptors and the late phase is predominantly dependent on inflammatory mediators [24]. 5-CK was able to inhibit both early and late phases, a profile similar to that of morphine, indicating it could be acting as a pure analgesic. Indeed, central analgesics, like morphine and tramadol, that inhibit the pain signal transmission are able to inhibit both early and late phases of the formalin test [25]. Interestingly, the nociceptive behavior on the inflammatory phase was greatly reduced. This could be due to the combined inhibition of pro-inflammatory mediators, a previously demonstrated effect of 5-CK [18] and modulation of nociceptive input. Reinforcing this idea, aminopyrine and mefenamic acid, compounds with a central and a peripheral site of action, showed an antinociceptive effect on both phases, but the second phase response was inhibited by lower doses [26], as observed here. In order to investigate this hypothesis, the involvement of a central component in the 5-CK antinociception was next evaluated.

For this proposal, cold plate and tail flick tests, both thermic models able to detect centrally acting analgesics, were used. The tail flick test is considered a spinal reflex, but could also involve higher neural structures [27], while the nociceptive behaviors of the cold plate test are considered integrated responses at the supraspinal level [28]. These tests are therefore widely used in the characterization of centrally acting analgesics. 5-CK increased response latency on the tail flick test and consistently reduced the nociceptive behavior on the cold plate test, thus, indicating that its analgesic effect is mediated by a central component. The observed antinociceptive effect was dose-dependent and, importantly, it was longer-lasting and more efficacious than that induced by morphine, the gold standard painkiller drug. 5-CK inhibited the nociceptive behavior on cold plate test for up to 8 h, which is consistent with previous reports of its high metabolic stability [18], a property of interest for drug development.

Next, aiming to understand how 5-CK exerts this antinociceptive effect, a functional antagonism assay was employed. Opioid receptors and their endogenous ligands are widely distributed throughout the central nervous system, and for that reason are the most explored pathway for pain modulation [29]. Based on the morphine-like antinociception profile seen in nociceptive tests, and the fact that opioid ligands are the primary mediators of central analgesia, the involvement of 5-CK with the opioid system was investigated first. The pre-treatment with the opioid antagonist naloxone did not prevent the antinociceptive effect of 5-CK on both tail flick and cold plate tests, which implies that 5-CK does not interact with opioid receptors to produce its effect. Although opioid ligands are the primary neurotransmitters involved within the endogenous analgesia system, pain has different components and a great number of neurotransmitters can modulate the nociceptive input and, therefore, be targets for pharmacological modulation. Therefore, other pathways and receptors, such as α-adrenergic, serotonergic, cholinergic, GABAergic, nitrergic, and K^+^_ATP_ channels, which play an important role in the neural pathways of conduction, processing, and modulation of pain [29,30] may be involved in 5-CK antinociception.

Given the large number of pathways and neurotransmitters involved with pain modulation, an in silico approach was used to highlight putative targets of 5-CK. To that end, a model was proposed comparing the 2D and 3D descriptors of 5-CK to that of antagonists of key pain pathways, assuming that structurally similar compounds have similar physicochemical properties and, consequently, similar biological targets [31,32,33,34,35,36,37]. Tanimoto index is often employed for this task since values >85% imply a similar mechanism of action [34]. The low values obtained in this analysis would suggest that 5-CK binds to a different target than the antagonists related to common analgesia pathways. However, there are cases where compounds with low 2D similarity still bind to the same target, because they display high 3D similarity [38]. The morphological similarity analysis, carried out in this work, underscores a similar trend, as 5-CK is predicted to have similar shape and electrostatic/hydrophobic features to atropine, a cholinergic antagonist, bicuculline, a GABA_A_ channel antagonist, and glibenclamide, a K^+^_ATP_ channel blocker. Therefore, testing efforts were next focused on assessing the participation of these pathways on the 5-CK-induced antinociception. The pharmacological blockade of muscarinic receptors or K^+^_ATP_ channels did not alter 5-CK antinociception, indicating that they do not contribute to 5-CK’s mechanism of action. Conversely, the blockade of GABA_A_ receptors abolished the 5-CK antinociceptive effect, indicating that activation of GABAergic receptors is important for this antinociception. In fact, the interplay of chromone derivatives with the GABAergic system has been previously described. A chromanone derivative isolated from *Hypericum lissophloeus* was found to potentiate GABA_A_ receptor currents [39], whereas the flavonoid viscosine is also a positive modulator of GABA_A_ receptors [40]. In addition, quercetin and other dimethoxy flavones had their antinociceptive effect mediated by GABAergic pathways [10,41].

GABA and GABAergic neurons are present throughout the pain pathway in the central nervous system, in supraspinal regions that regulate endogenous analgesia systems, and in the dorsal horn of the spinal cord, a key site of pain transmission [42]. Systemic administration of GABAergic agonists generally produces antinociception in animal models of acute and persistent pain [43]. As GABA_A_ receptors are involved in the modulatory processing of nociceptive input [44] these receptors have been considered a potential pharmacological target for the treatment of pain. This concept has been reinforced by preclinical [25,45] and clinical studies [46].

The development of new non-opioid analgesics is pivotal to approaching many painful states in which opioids are not effective. Some peripheral neuropathies, for instance, have in general a poor response to opioids. On the other hand, substances that act on GABAergic pathways, chromones included, have shown preclinical efficacy on different types of neuropathy, making them a potential source of relief to these painful conditions [11,12,45,47,48].

In this study, the involvement of GABAergic pathways with the antinociceptive activity of 5-CK was demonstrated, but this effect can be mediated by different types of interaction within the GABA_A_ receptor. The GABA_A_ receptor function, and finally its physiologic effect, can be modified both by agents that, such as bicuculline, interact directly with the receptor recognition site (orthosteric sites), and by agents that interact with sites other than the GABA ligand-binding domain (allosteric sites), such as benzodiazepines, barbiturates and neurosteroids [42,49]. Effectively, several GABA_A_ selective allosteric modulators exert antinociceptive activity on animal models of neuropathic pain [48,50,51,52] and pain conditions on human volunteers [53,54,55,56].

Although nociceptive tests evidence the involvement of GABA_A_ receptor on the 5-CK-induced antinociception, and in silico results suggest that 5-CK binds to the orthosteric binding site, neither the steric, nor the electronic features of the GABA_A_ binding site were taken into account to support or oppose this hypothesis up to this point. Docking studies were undertaken to fill this knowledge gap. According to this analysis, 5-CK displays a similar binding profile to that observed for bicuculline and GABA (Supplementary Materials Figure S4). First, the hydrophobic nature of chromone and dihydropyran rings of the 5-CK enable its interaction with “aromatic box” residues [57] and this seems to be a common interaction motif for several GABA receptor ligands such as bicuculline and gabazine [58]. In addition, the 2-penten-4-ol moiety is predicted to interact with Arg67B, as has been observed for muscimol and 4-PIOL analogs [59]. This interaction is expected to anchor the carboxyl group of GABA [60], the benzodioxole of bicuculline, and it is predicted to be important for several GABA analogs, such as THIP and muscimol [61]. Last but not least, the carbonyl from the chromone core is predicted to make a hydrogen bond to Try97A. GABA displays a similar interaction to this [57,61].

In conclusion, the present study showed that the natural chromone 5-CK possesses intrinsic antinociceptive properties associated with central nervous system modulation. Moreover, by using in silico approaches a light was shed on the mechanism of action of this chromone. Using functional antagonism assays, the involvement of GABA_A_ receptors in the antinociceptive activity of 5-CK was demonstrated. Modeling also points to the GABA binding site as a specific binding site to 5-CK within the GABA_A_ receptor. These results present 5-CK as a prototype of a novel multimodal non-opioid analgesic that could be used on pain states when the therapy with opioids is neither safe nor effective. Plus, the different scaffold opens a perspective to the synthesis of novel chromone compounds with analgesic properties. Despite the evidence presented in this work, additional binding studies should be carried out to confirm the exact binding site of 5-CK on GABA_A_ receptors.

## 4. Materials and Methods

### 4.1. Animals

Experiments were performed on male Swiss Webster mice obtained from the Animal Facilities at Instituto Gonçalo Moniz (Salvador, Brazil). Animals (24–28 g) were housed in temperature-controlled rooms (22–25 °C), under a 12:12 h light-dark cycle, with access to water and food ad libitum until experimental initiation. All behavioral tests were performed between 8:00 a.m. and 5:00 p.m. Animal care and handling procedures were in strict accordance with the recommendations in the Guide for the Care and Use of Laboratory Animals of National Institute of Health and Brazilian College of Animal Experimentation. The protocol was approved by the Institutional Animal Care and Use Committee of FIOCRUZ (CEUA/FIOCRUZ, permit number: L-IGM-015/2013, approved at 23 December 2013). Every effort was made to minimize the number of animals used and any discomfort. Behavioral tests were performed without knowing to which experimental group each mouse belonged.

### 4.2. Extraction and Isolation

5-*O*-methylcneorumchromone K (5-CK) was isolated from the root bark of *Dictyoloma vandellianum* (Rutaceae) collected in March 2005 in Piatã, Brazil (13°140 43″ S, 41°450 28″ W). The plant was identified by Dr. Maria Lenise Silva Guedes from the Herbarium Alexandre Leal Costa of the Federal University of Bahia, Brazil. A voucher specimen (no. 69163) has been deposited at the Herbarium Alexandre Leal Costa. The procedures used for the purification of 5-CK have been described [62]. The percent purity of 5-CK used in the pharmacological experiments carried out was greater than 98%, as determined by high performance liquid chromatography.

### 4.3. Formalin Test 

Mice were placed in an open Plexiglas observation chamber for 30 min to acclimate to their surroundings and then removed for formalin administration. Mice were gently restrained while 20 μL of 2.5% formalin (1:100 dilution of stock formalin solution, 37% formaldehyde in 0.9% saline) was administered subcutaneously to the dorsum of the hind paw using a 30-gauge needle. Following injection, mice were returned to the observation chamber for a 30 min observation period. A mirror was placed behind the chamber to enable unhindered observation of the formalin-injected paw. Mice were observed from 0 to 10 min (early phase) and from 10 to 30 min (late phase), and a nociception score was determined for each period by counting the time that the animal spent licking the injected limb during the observation time [63]. Mice were treated with 5-CK (0.78–50 mg/Kg), vehicle (50% propylene glycol in saline; control group), indomethacin (10 mg/Kg, reference drug), or morphine (5 mg/Kg, reference drug) by intraperitoneal (ip.) route 40 min before formalin administration. Indomethacin and morphine were purchased from Cristália (Itapira, São Paulo, Brazil).

### 4.4. Motor Function Assay: Rota-Rod Test

To evaluate a possible interference in the motor performance, mice were submitted to the rota-rod test, as previously described [18]. The rota-rod apparatus (Insight, Ribeirão Preto, Brazil) consisted of a bar with a diameter of 3 cm, subdivided into five compartments. Mice received intraperitoneal administration of chromones (100 mg/Kg) or diazepam (10 mg/Kg), used as the positive control, and 40 min afterward were placed on a rotating rod (6 rpm) and the falling avoidance was measured for up to 120 s. The results were analyzed as the average time (s) that the animals remained on the rota-rod in each group. Diazepam was purchased from Cristália (Itapira, São Paulo, Brazil).

### 4.5. Tail Flick Test

The tail flick test in mice was conducted as described elsewhere [63]. The day before the experiment, each animal was habituated to the restraint cylinder for 20 min/day for 5 consecutive days. On the day of the experiment, mice were placed in the restraint cylinder, and the tail tip (2 cm) was submerged in a water bath at 48 ± 0.5 °C. The latency of the tail withdrawal reflex was measured. Each submersion was terminated after 10 s to minimize potential skin damage. Tail flick latency was measured before (baseline) and after treatments. Mice were treated with 5-CK (6.25–50 mg/Kg), vehicle (50% propylene glycol in saline; control group), or morphine (5 mg/Kg, reference drug) by ip. route.

### 4.6. Cold Plate Test

The cold thermal nociceptive threshold was evaluated in a cold plate device (Teca^®^, Chicago, IL, USA) at a temperature of −2.5 ± 0.2 °C. The animals were kept for five minutes on the cold plate and the nociceptive response was quantified by counting the nociceptive behaviors namely, hind paw lifts, hind paw lickings, flinches, and jumps [64]. The animals were acclimated to the cold plate the day before the test, remaining for 2 min at the same temperature used in the test. The results were expressed as nociception index, which stands for the total amount of nociceptive responses in 5 min.

### 4.7. Functional Antagonism Assays

Further experiments were carried out to elucidate the possible mechanisms by which 5-CK exert its antinociceptive action. Antagonism assays were conducted employing tail flick and cold plate tests, using the maximum effective dose of 5-CK (50 mg/Kg). Mice were intraperitoneally pretreated with antagonists 15 min or 30 min prior to the administration of 5-CK. The antinociceptive response was recorded 40 min after treatment with 5-CK. The following antagonists were tested: non-selective opioid antagonist, naloxone (5 mg/Kg, 15 min before) [30]; ATP-sensitive potassium channel blocker, glibenclamide (2 mg/Kg, 30 min before) [65]; gamma aminobutyric acid-A GABA_A_ receptor antagonist, bicuculline (1 mg/Kg, 15 min before) [30]; cholinergic receptor antagonist, atropine (10 mg/Kg, 15 min before) [30]. The tested drugs were purchased from Sigma-Aldrich (St Louis, MO, USA) and naloxone was purchased from Cristália (Itapira, São Paulo, Brazil).

### 4.8. Chemical Similarity Analysis

The chemical similarity of 5-CK to known ligands of receptors and channels related to endogenous analgesia pathways (methysergide maleate —serotonergic receptor antagonist; yohimbine—alpha-2 adrenergic receptor antagonist; atropine—cholinergic receptor antagonist; bicuculline—gamma aminobutyric acid-A receptor antagonist—GABA_A_; phaclofen—gamma aminobutyric acid-B receptor antagonist—GABA_B_; L-arginine—a precursor of nitric oxide; glibenclamide—ATP-sensitive potassium channel blocker) [30,65] was assessed using 2D and 3D descriptors. First, all molecules were drawn in the Marvin^®^ Sketch 19.1 software (Chemaxon, Budapest, Hungary). Then, for the 2D analysis, atomic descriptors were calculated for pairs of atoms in each molecule based on the atoms types, the shortest length between the atoms, the number of their pi electrons and the non-hydrogen atoms attached to them [66]. Common and unique paths (fingerprints) were counted for each molecule and then used to compute the Tanimoto similarity coefficient [67,68,69], as available on the ChemMine tools server (http://chemminetools.ucr.edu/). The similarity ranges from 0.0 (completely different) to 100.0% (equal molecules). Molecules in 2D format were then converted to the 3D format using the Concord module (translate molecular files with the standard parameters) available on the Sybyl^®^-X 2.1.1 (Tripos, 2013), had their Gasteiger-Huckel charges [70,71] calculated (ε = 80.4) and then were energy minimized by conjugate gradient (convergence criteria = 0.001 Kcal/mol), using Tripos force-field [72], available in Sybyl^®^-X 2.1.1. The lowest energy conformation of 5-CK was employed as a template for morphological similarity analysis, using standard parameters from Surflex-Sim module, available on Sybyl-X 2.1.1. Briefly, known ligands (queries) were flexibly aligned to 5-CK, aiming at maximizing the molecule’s shape overlap, so that hydrogen bonding and electrostatic interaction between the template and each query are similar. The morphological similarity score (MSS) ranges from 0.0 (completely different) to 10.0 (equal 3D shape and interaction profile) [73].

### 4.9. Docking Studies: Ligands and 3D Protein Structure Preparation

The Cryo-EM 3D structure of α_1_β_3_γ_2_ GABA_A_ receptor complexed with gamma aminobutyric acid (PDB: 6X3T) was used for docking studies. First, the atomic coordinates of protein were morphed to fit the coordinates of GABA_A_ receptor complexed with bicuculline (PDB: 6HUK), using default parameters from Morphit Pro server [19], except for the number of frames that was adjusted to 30. The morphed 3D structure (frame 25) was then prepared for docking search using the Biopolymer module available on Sybyl^®^-X 2.1.1. H-bond optimized hydrogens were then added and histidine, glutamate and aspartate residues were manually checked for flip orientation, protonation and tautomeric states. The protonation state of residues was estimated at physiological pH (7.4) using the Propka 3.1 server (https://server.poissonboltzmann.org/) [74,75]. Finally, “AMBER Force Field 99” charges [76] were assigned to all protein residues. The energy-minimized structures of bicuculline and 5-CK were prepared as described in the chemical similarity analysis section.

### 4.10. Molecular Docking Studies

Conformational search and evaluation of the ligand poses were carried out in the GOLD 2020.2 software (CCDC, Cambridge, UK) [77,78,79,80]. The search space was set with the residues within 10Å radius from the center of bicuculline. The protein residues were kept rigid throughout the calculations, while the ligands were held flexible, including N-pyramidal and ring-corners. Docking calculations were performed using a Lamarckian genetic algorithm (LGA) [81] and search efficiency settings adjusted to very flexible (60,000 LGA operations). The three best-ranked poses, according to the Piecewise Linear Potential score function (ChemPLP) [82,83], had their interaction profile calculated with Poseview (http://poseview.zbh.uni-hamburg.de/) and PLIP (https://projects.biotec.tu-dresden.de/plip-web/plip) servers and, after that, were visually inspected in Pymol software version 1.8 (New York, NY, USA), to discard biologically irrelevant poses (i.e., a hydroxyl group buried in a hydrophobic pocket).

### 4.11. Statistical Analysis

Data are presented as means ± standard error of the mean (SEM) of measurements made on 6–9 animals in each group. Comparisons between three or more treatments were made using one-way ANOVA with Tukey’s post hoc test, or for repeated measures, two-way ANOVA with Bonferroni’s post hoc test, as appropriate. All data were analyzed using Prism 5 Computer Software (GraphPad, San Diego, CA, USA). Statistical differences were considered significant at *p* < 0.05.

## 5. Conclusions

The present work described for the first time the intrinsic antinociceptive properties of the natural chromone 5-CK. The administration of 5-CK produced a long-lasting and dose-dependent antinociceptive response on cold plate and tail flick tests. Functional antagonism assays provided evidence of the involvement of GABA_A_ receptors with the antinociceptive activity of 5-CK. The results also reveal that opioid receptors, muscarinic receptors or K^+^_ATP_ channels do not participate on the 5-CK-induced antinociception. Plus, the results from the in silico analysis also suggest that 5-CK act by interacting with the GABA binding site at the GABA_A_ receptor.

## Data Availability

The data presented in this study are openly available in Mendeley Data at <http://dx.doi.org/10.17632/8zwjchfkw7.1>.

## Supplementary Materials

The following are available online at https://www.mdpi.com/article/10.3390/ijms22073413/s1. Figure S1: Two-dimensional (2D) structures of 5-CK and several antagonists of receptors and ion channels related to endogenous analgesia pathway. Figure S2: Bicuculline binding site highlighting the conformational change of residues. Figure S3: Bicuculine re-docking pose in relation to Cryo-EM bicuculline position on GABA_A_ receptor structure. Figure S4: Interaction profile of Bicuculline and Gama amino butyric acid on GABA binding site generated by Poseview server.

## Abbreviations

5-CK	5-*O*-methylcneorumchromone K
GABA	Gamma aminobutyric acid
NSAID	non-steroidal anti-inflammatory drugs
ANOVA	analysis of variance
ip.	intraperitoneal
CTRL	control group
SEM	standard error of the mean
PDB	Protein Data Bank
PLIP	Protein-Ligand Interaction Profiler
RSMD	root-mean-square deviation
K^+^_ATP_	ATP-sensitive potassium channel
4-PIOL	(3-hydroxy-5-(4-piperidyl) isoxazole)
THIP	4,5,6,7-tetrahydroisoxazolo[5,4-c] pyridin-3-ol
FIOCRUZ	Oswaldo Cruz Foundation
Cryo-EM	Cryogenic electron microscopy
