# Supplementary material for: 5-O-methylcneorumchromone K Exerts Antinociceptive Effects in Mice via Interaction with GABAA Receptors
Source: Int J Mol Sci. 2021 Mar 26;22(7):3413. doi: 10.3390/ijms22073413 (PMC8037321; doi:10.3390/ijms22073413)
Supplement: Supplementary file 1 [file ijms-22-03413-s001.pdf]

Supplementary Material

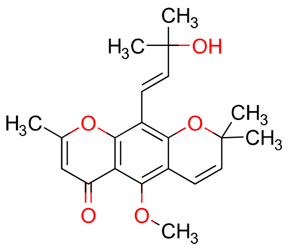

5-CK

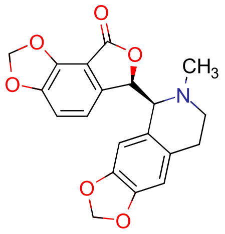

Bicuculline

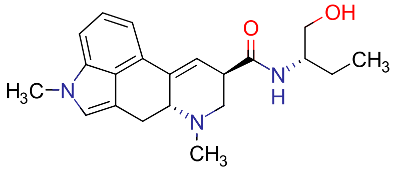

Methysergide

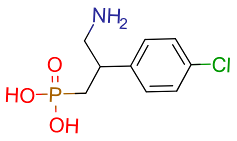

Phaclofen

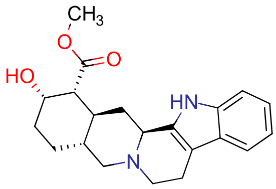

Yohimbine

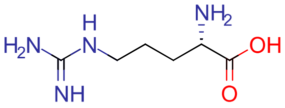

L-arginine

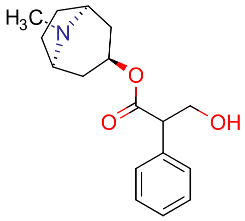

Atropine

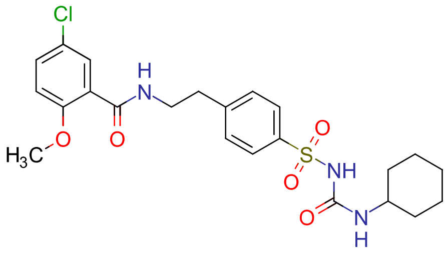

Glibenclamide

Figure S1 - Two-dimensional (2D) structures of 5-CK and several antagonists of receptors and ion channels related to endogenous analgesia pathway: methysergide; yohimbine; atropine; bicuculline; phaclofen; L-arginine; and glibenclamide.

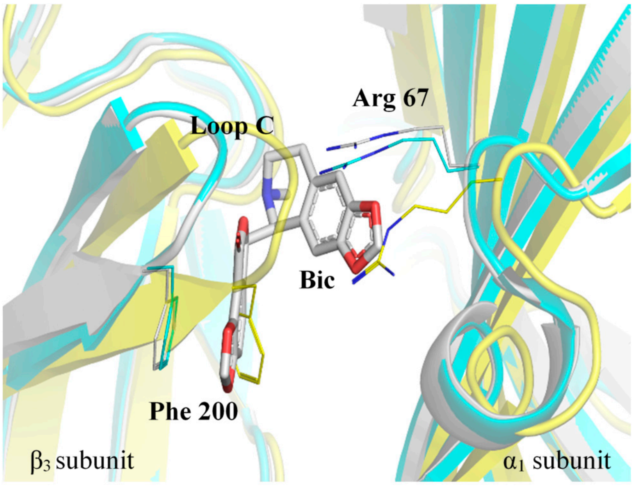

Figure S2 - Bicuculline binding site highlighting the conformational change of residues Phe 200 (β_3) and Arg 67 (α_1) in bonded (PDB: 6HUK, white) and unbonded (PDB: 6T3X, yellow) GABA$_A$ receptor with bicuculline. The selected morph (frame 25) was colored in cyan.

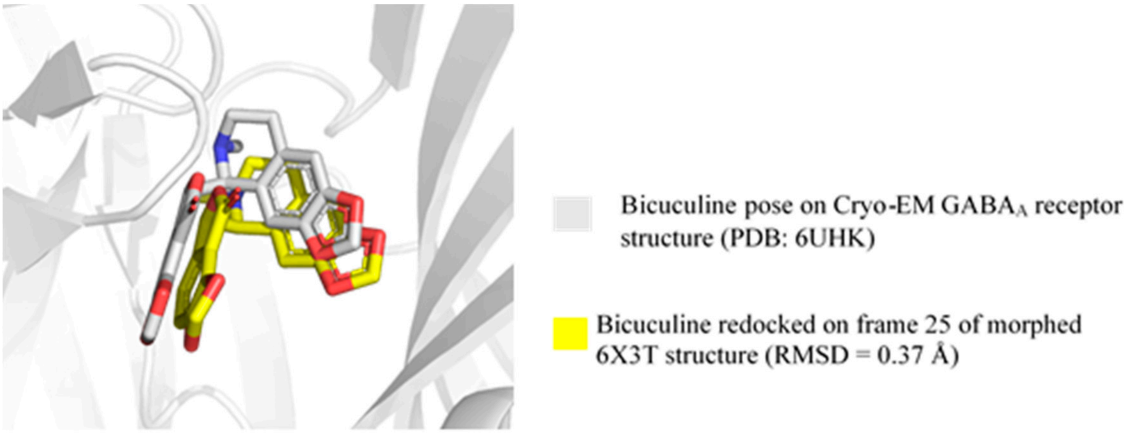

Figure S3 – Bicuculine re-docking pose (yellow) in relation to Cryo-EM bicuculline position (white) on GABA$_A$ receptor structure.

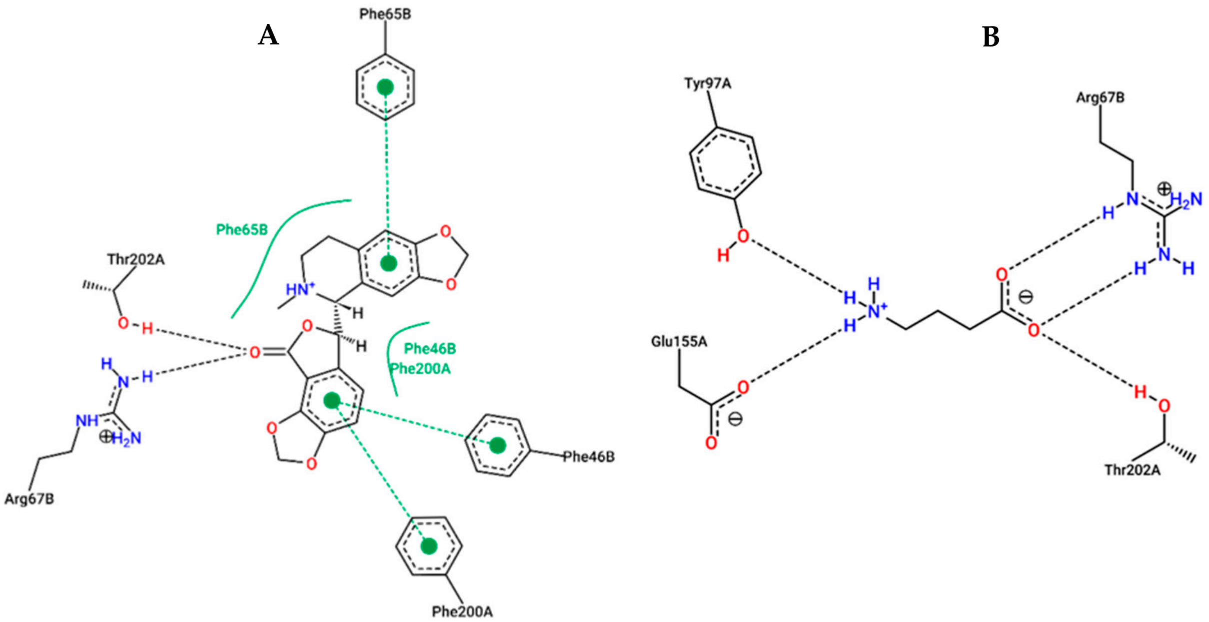

Figure S4 - Interaction profile of Bicuculline; (**A**) and Gama amino butyric acid; (**B**) on GABA binding site generated by Poseview server.
